# Peer Review of “The Influence of SARS-CoV-2 Variants on National Case-Fatality Rates: Correlation and Validation Study”

**DOI:** 10.2196/38519

**Published:** 2022-05-24

**Keywords:** SARS-CoV-2, COVID-19, variants of concern, case fatality rates, virulence, vaccine effectiveness, correlation study

*This is a peer-review report submitted for the paper “The Influence of SARS-CoV-2 Variants on National Case-Fatality Rates: Correlation and Validation Study.”*

## Round 1 Review

### General Comments

This paper [1] used ecological data to study the correlation between SARS-CoV-2 variants and the fatality rates. It introduced a new indicator to correct for the lagging of the reported death since the initial infection. When applying this indicator to different countries, it demonstrated that the spreading of variants coincided with the surge in death while also acknowledging the potential confounding factors such as vaccination rates. Although the conclusions drawn in this paper showed some inconsistency with other observational/​community-based epidemiological studies, the paper also explored the correlation between disease risk factors and the reported death.

### Specific Comments

#### Major Comments

1. The author should provide more characterizations of the proxy case-fatality rate (pCFR). For example, the author should compare the pCFR and the case-fatality rate (CFR) while doing the analysis, such as correlation analysis.

2. The author mentioned “One could equally well average the infection rate over the period from 28 to 14 days,” but no figure was also presented. Comparing different parameters used to construct the pCFR is essential for the reader to evaluate the robustness of the proposed indicator.

3. Related to the first point, the author should probably also compare the raw CFR 7-day rolling average and the pCFR 7-day rolling average.

4. The death rate is also related to the capacity of the health care system, such as available intensive care unit (ICU) facilities or bed occupancy. Thus, the CFR on a particular day might also depend on the CFR (as an approximation to the ICU occupancy) the day before. While the author reported the absolute pCFR percentage in most of the figures, these results should also be confirmed by replotting the percentages as relative percentages. For example, one could report the daily pCFR as the percentage change to the previous day (or the previous 7-day rolling average).

5. By doing point 4 above, the relative pCFR can be used to compare different included countries that have daily CFRs that are highly variable.

6. The risk factor correlation analysis can be misleading. The author should state very clearly that ecological data were used for the analysis, both in the Introduction and Discussion sections. It has been shown that a population-based correlation provided little insight into understanding the disease pathology. (Portnov B, Dubnov J and Barchana M. On ecological fallacy, assessment errors stemming from misguided variable selection, and the effect of aggregation on the outcome of epidemiological study. J Expo Sci Environ Epidemiol 2007; 17:106-121).

7. It is unclear that the definitions of each of the variables (risk factors) are included in the correlation analysis. While I assume it is the same as those cited in the second reference, some of the analysis methodologies seem imprecise. For example, epidemiologists usually model the age as ordinary variables and test for the trend (eg, using ANOVA) but not by using the median age. The author might want to revisit some of the analyses performed.

8. As the author also pointed out, many of these risk factors are correlated with each other. A better way to adjust for these potential confounding effects is by modeling all these risk factors in a regression model.

9. The author should explain the choice of “shift by 60 days” in Figure 12.

### Minor Comments

10. The author should consider unifying the color scheme used in the manuscript. For example, some figures are plotted in grayscale, but similar figures can also appear in a colored version.

11. In equation 2, “Total cases on day (N-14) - Total cases on day (N-21),” the “-” between the two phrases can be misleading. The author should consider rewriting the “-” as “to.”

12. The author should also consider replotting the correlation analysis into heat maps. The author did not justify the use of a line plot for plotting each risk factor.

13. Furthermore, the author should consider clustering the risk factor and plotting a dendrogram with the heat map. Therefore, it will give readers a better idea of the correlation among each risk factor and the correlation among each of the cutoff dates (in Figure 6) or regions (in Figure 7).

## Round 2 Review

This draft has been greatly improved but the author should still consider the following:

1. Rewrite the denominator of equation 11 using the summation sign

2. In the current manuscript, equation 2 appeared before equation 1.

3. There were multiple equation 2s. Equation 1 also appeared twice: in the main text and in the supplementary text.

4. It is better to always mention the year for the date/period that was referenced in the manuscript (eg, “B.1.1.7 (Alpha) and B.1.351 (Beta) strains dated from mid-October and mid-May respectively” and “that could be due to masking by the fraction of Delta cases peaking in Argentina in mid-May” in the Result section).

5. The meaning of the statement “The positive aspect of that limitation is that trends in pCFR can spot burn through cases in unvaccinated of less than vigilant groups” is unclear.

6. The author mentioned “The red points are due to anomalous entries in the tables of (13)” in the Result section. It would be better to clean the data for the suspected anomalous entries mentioned in the Methods section while plotting the smoothened graph.

7. Regression results should be listed in tables that show (at least) effect size and *P* value.

## Abbreviations

CFR: case-fatality rate
ICU: intensive care unit
pCFR: proxy case-fatality rate
